# *Tsc1* ablation in Prx1 and Osterix lineages causes renal cystogenesis in mouse

**DOI:** 10.1038/s41598-018-37139-9

**Published:** 2019-01-29

**Authors:** Zhixiang Wu, Hongguang Wu, Shafiquzzaman Md, Guo Yu, Samy L. Habib, Baojie Li, Jing Li

**Affiliations:** 1 Bio-X Institutes, Key Laboratory for the Genetics of Developmental and Neuropsychiatric Disorders, Ministry of Education, Shanghai Jiao Tong University, Shanghai, 200240 China; 2 Department of Cellular and Structural Biology, South Texas Veterans Health Care System, San Antonio, University of Texas Health Science Center at San Antonio, San Antonio, TX 78229 USA; 3 Department of Ophthalmology, XinHua Hospital, Shanghai Jiao Tong University School of Medicine, Shanghai, China

## Abstract

Tuberous Sclerosis Complex (TSC) is caused by mutations in *TSC1* or *TSC2*, which encode negative regulators of the mTOR signaling pathway. The renal abnormalities associated with TSC include angiomyolipoma, cysts, and renal cell carcinoma. Here we report that specific ablation of *Tsc1* using the mesenchymal stem cell-osteoblast lineage markers induced cystogenesis in mice. Using *Rosa-tdTomato* mice, we found that Prx1- or Dermo1-labeled cells were present in the nephron including glomerulus but they were not stained by markers for podocytes, mesangial cells, endothelial cells, or proximal or loop of Henle tubular cells, while Osx is known to label tubular cells. *Tsc1* deficiency in Prx1 lineage cells caused development of mild cysts that were positive only for Tamm-Horsfall protein (THP), a loop of Henle marker, while *Tsc1* deficiency in Osx lineage cells caused development of cysts that were positive for Villin, a proximal tubular cell marker. On the other hand, *Tsc1* deficiency in the Dermo1 lineage did not produce detectable phenotypical changes in the kidney. Cyst formation in *Prx1-Cre*; *Tsc1*^*f/f*^ and *Osx-Cre*; *Tsc1*^*f/f*^ mice were associated with increase in both proliferative and apoptotic cells in the affected tissue and were largely suppressed by rapamycin. These results suggest that Prx1 and Osx lineages cells may contribute to renal cystogenesis in TSC patients.

## Introduction

Tuberous sclerosis complex (TSC) is an autosomal dominant genetic disease caused by mutations in *TSC1* or *TSC2*. TSC patients usually display overgrowth in brain, kidney, lung, eye, and skin^1–3^. If left untreated, nearly 40% of TSC patients die by the age of 35 years, mainly due to renal failure or hemorrhage^4^. In kidney, TSC symptoms include angiomyolipoma, cysts, and rarely renal cell carcinoma. Angiomyolipomas are of mesenchymal origin and composed of varying proportions of vascular cells, smooth muscle cells, and fat cells, whereas cysts are of epithelial origin^5^. Genetic ablation of one allele of *Tsc1* or *Tsc2* in mouse recapitulates the phenotypes of TSC patients^6^.

*Tsc1* and *Tsc2* encode two negative regulators of the mTOR signaling pathway. mTOR signaling plays important roles in the development and homeostasis of multiple tissues and organs. The mTOR pathway consists of mTOR complex 1 (mTORC1) and mTOR complex 2 (mTORC2). mTORC1 is composed of mTOR and regulatory associated protein of mTOR (Raptor), while mTORC2 is composed of mTOR and rapamycin independent companion of mTOR (Rictor). Rapamycin-sensitive mTORC1 activation promotes cell proliferation and growth in size. Rapamycin-insensitive mTORC2 controls the cytoskeleton and cell shape. Growth factors activate the PI3K-Akt pathway, which deactivates the heterodimer complex of TSC1 and TSC2, resulting in activation of mTORC1 through Ras-related small GTPase (Rheb). Activated mTORC1 promotes transcription and protein translation to promote cell proliferation and growth via phosphorylation of both p70S6K and eukaryotic initiation factor 4E-binding protein 1 (4E-BP1). Brook-Carter *et al*. in 1994 discovered that deletion of *TSC2* was associated with severe infantile polycystic kidney disease^7^. Later, a series of reports suggested that misregulation of mTOR signaling would produce polycystic kidney disease^8–12^. In TSC animal models, renal cysts and cell hyperproliferation are detected. Pema. *et al*. reported that mTORC1-mediated inhibition of polycystin-1 expression might underlie renal cyst formation^13^. On the other hand, a recent study suggested that TSC-induced tumorigenesis was independent of mTOR pathway activation^14^.

Kidney growth is regulated by mTOR signaling^15^. The main structure and function units of kidney are the nephrons, which are composed of blood-filtrating renal corpuscle and renal tubules. The renal corpuscle is made of glomerulus and the surrounding Bowman’s capsule. It is estimated that renal tubular cells may have 16 different types of epithelial cells. Developmentally, kidney is derived from the intermediate mesoderm. While Wolffian duct-derived epithelial cells form the ducts, Six2-positive mesenchymal cells form the nephrons and Foxd1-positive cells give rise to the stromal cells in the kidney^16^. Both Six2-positive nephrons and Foxd1-positive interstitial cells are derived from Osr1-positive progenitor cells prior to embryonic day (E) 10.5^17^.

In this study, we showed that two mesoderm-derived mesenchymal stem cell (MSC) markers, *Prx1* and *Dermo1*, could label previously unidentified renal stromal cells. A previous study indicated that Osterix (Osx), a transcription factor essential for osteoblast differentiation, labeled renal tubular cells^18–20^. We deleted *Tsc1* in Prx1, Dermo1, and Osx positive cells using corresponding Cre mouse lines and found significant abnormalities in the kidneys of *Prx1-Cre; Tsc1*^*f/f*^ and *Osx-Cre; Tsc1*^*f/f*^ mouse lines but not *Dermo1-Cre; Tsc1*^*f/f*^ mice. *Prx1-Cre; Tsc1*^*f/f*^ mice had cysts derived from loop of Henle, whereas *Osx-Cre; Tsc1*^*f/f*^ mice had cysts formed from proximal tubules. The overgrowth phenotypes were likely caused by increased cell proliferation, which could be rescued by intraperitoneal injection of rapamycin.

## Result

### Lineage tracing identified Prx1-expressing cells in mouse kidney

MSCs harbor the potentials to differentiate into many cell types, especially osteoblasts, chondrocytes, and adipocytes. *Prx1* and *Dermo1* are generally used as genetic marks for MSCs in skeletal studies^21–24^. We have previously established *Tsc1* knockout mice using *Prx1-Cre*, *Dermo1-Cre*, and *Osx-Cre* mouse lines to study mTOR signaling in skeletal growth and development^25^. Interestingly, we found that the kidneys of these mice also displayed some anomalies, suggesting that these markers may also label kidney cells.

**Figure 1:**
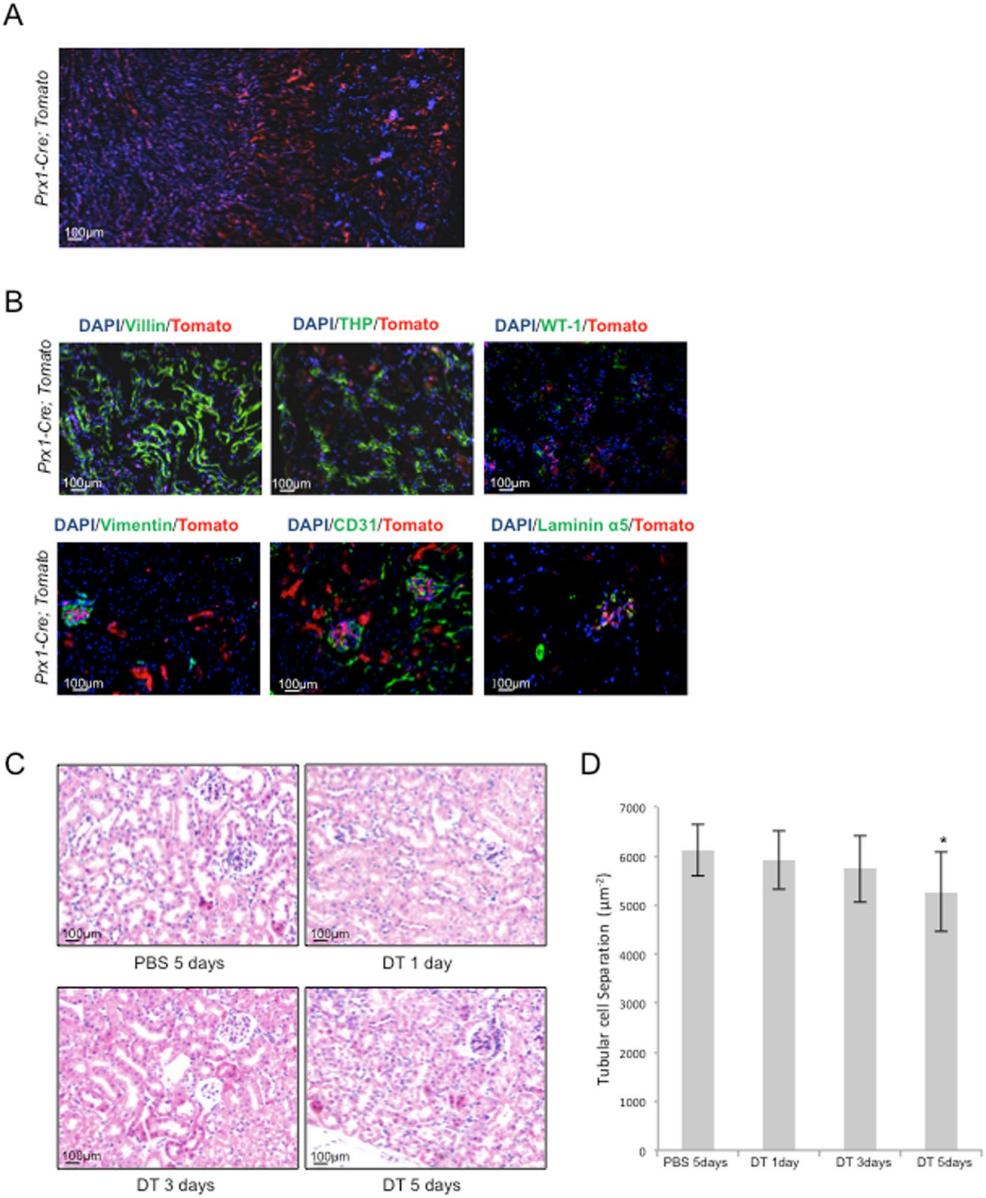
*Prx1* marks a population of cells in mouse kidney. (**A**) Microscopic images of kidney sections of *Prx1-Cre; tdTomato* mice. (**B**) Immunofluorescent staining on *Prx1-Cre; tdTomato* mouse kidney sections revealed that Prx1 linage renal cells were negative for the tested markers of different renal cell types. (**C**) DT was injected into *Prx1-Cre; iDTR* mice daily for different periods of time as indicated to deplete *Prx1* lineage cells. Moue kidneys were sectioned and stained with H-E. (**D**) Quantitative analysis of tubular cell numbers. N = 3. *P < 0.05; **P < 0.001 when compared to the control mice.

We first traced Prx1-expressing cells using *Prx1-Cre; Rosa-tdTomato* mice to study whether Prx1 lineage cells existed in the kidney. Fluorescent microscopy revealed Tomato-positive cells in the cortex and medulla including glomerulus and tubular regions in adult mice (Fig. 1A). Immuno-staining revealed that Prx1 lineage cells were negative for Villin, a proximal tubular cell marker; THP, a loop of Henle marker; CD31, an endothelial marker; WT-1, a podocyte marker; Laminin α5, an epithelial marker; and vimentin, a mesangial marker (Fig. 1B). These results suggest that Prx1 lineage constitute a group of stromal cells/fibroblasts in the kidney.

To validate these findings, we crossed *Rosa*-*iDTR* mice with *Prx1-Cre* mice to generate *Prx1-Cre; iDTR* mice, which could be used to deplete the Cre-expressing cells^26–28^. Diphtheria toxin (DT) was injected daily for 1, 3, and 5 days to *Prx1-Cre; iDTR* mice and *iDTR* mice. Although the gross structure of the kidney was not altered in DT-treated mutant mice (Fig. 1C), the number of glomerulus was significantly reduced. Quantitation of tubular cells separation (the ratio between the number of tubular nucleus and renal cortex area) revealed that the average tubular cell number declined as well (Fig. 1D). Taken together, the results indicate that Prx1 lineage cells were important component of the nephrons.

### Mice with *Tsc1* deletion in Prx1-expressing cells showed mild renal cysts

**Figure 2:**
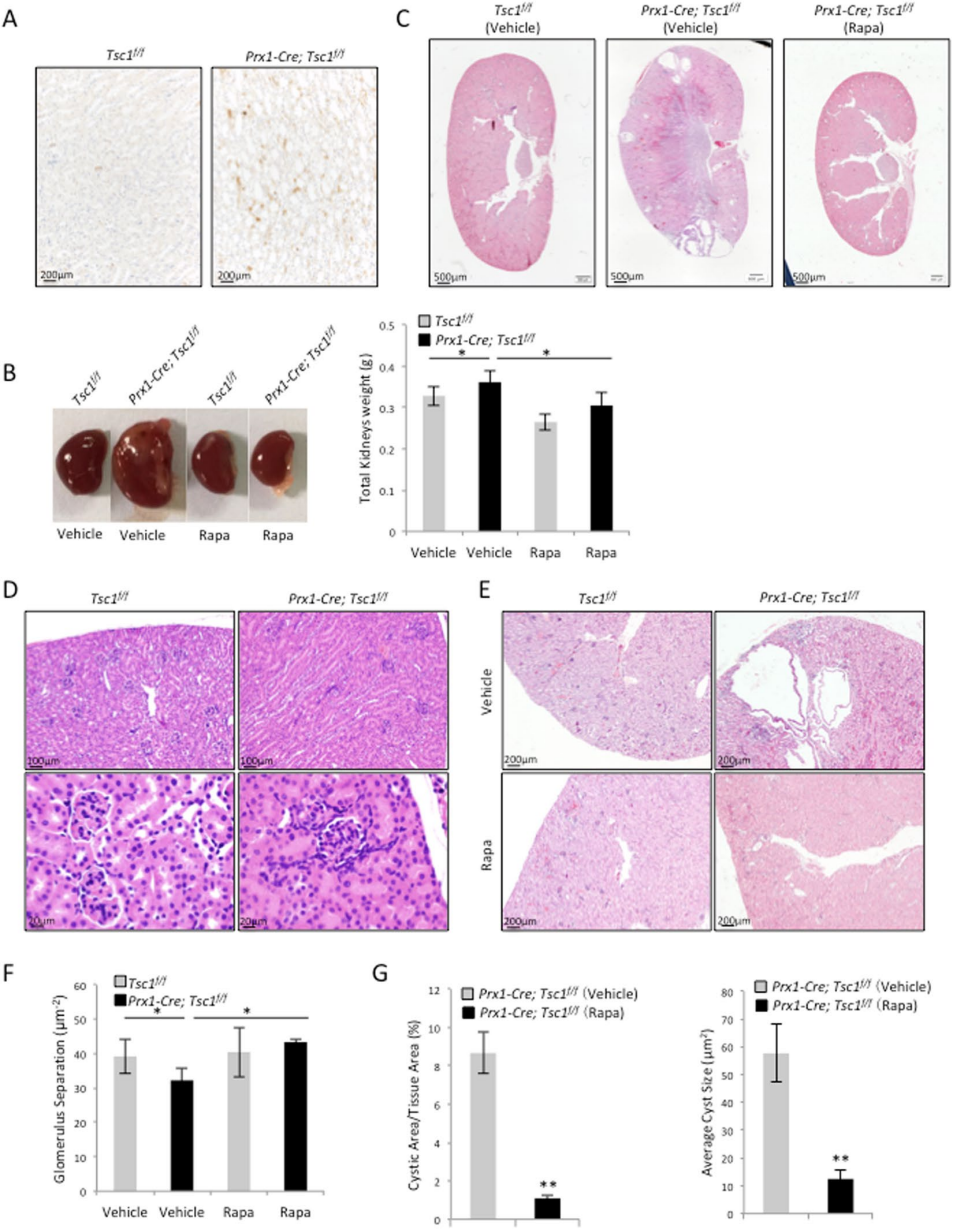
Conditional knockout of *Tsc1* in the Prx1 lineage resulted in the development of mTORC1-dependent cysts. (**A**) *Prx1-Cre; Tsc1*^*f/f*^ mouse kidney sections showed increased p-S6 signals. (**B**) *Prx1-Cre; Tsc1*^*f/f*^ mice showed enlarged kidneys that were rescued by rapamycin injection. Left panels: images of kidneys from different mouse lines. Right panel: the weights of the kidneys from different mouse lines. (**C**) H-E staining (40x) of the kidney sections of *Prx1-Cre; Tsc1*^*f/f*^ and control mice treated with rapamycin or vehicle. (**D**) H-E staining showed that 1- month-old mice did not develop cysts. (**E**) Images of kidney sections of *Prx1-Cre; Tsc1*^*f/f*^ and control mice treated with rapamycin or vehicle. (**F**) Cystic index of *Prx1-Cre; Tsc1*^*f/f*^ and control mice treated with rapamycin or vehicle. The cystic index for control mice is 0. (**G**) Quantitative analysis of glomerulus separation. N = 3. *P < 0.05; **P < 0.001 when compared to the control mice.

To understand the function of mTOR activation in Prx1 lineage cells of kidney, we crossed *Tsc1*^*f/f*^ mice with *Prx1-Cre* mice to generate *Prx1-Cre; Tscf1*^*f/f*^ mice. Immunohistological staining for p-S6, an indicator for mTOR pathway activation, revealed that ablation of *Tsc1* in Prx1 lineage cells resulted in an increase in mTOR activation in some of the renal cells (Fig. 2A). At the age of 2 months, the *Prx1-Cre; Tsc1*^*f/f*^ mice exhibited an increase in the size and weight of the kidneys compared to the control littermates (Fig. 2B). Histological examination revealed several cysts in the kidney (Fig. 2C), yet, no cyst was detected in 1-month-old mice (Fig. 2D), suggesting that the cysts were formed during mouse rapid growth period. While normal mice showed no cysts, the average cyst size and the percentage of cystic area occupying the total kidney cortex area indicated a mild polycystic kidney condition in the mutant mice (Fig. 2E). On the other hand, *Prx1-Cre; Tsc1*^*f/f*^ mice showed normal number and circumference of glomeruli (Fig. 2F,G).

**Figure 3:**
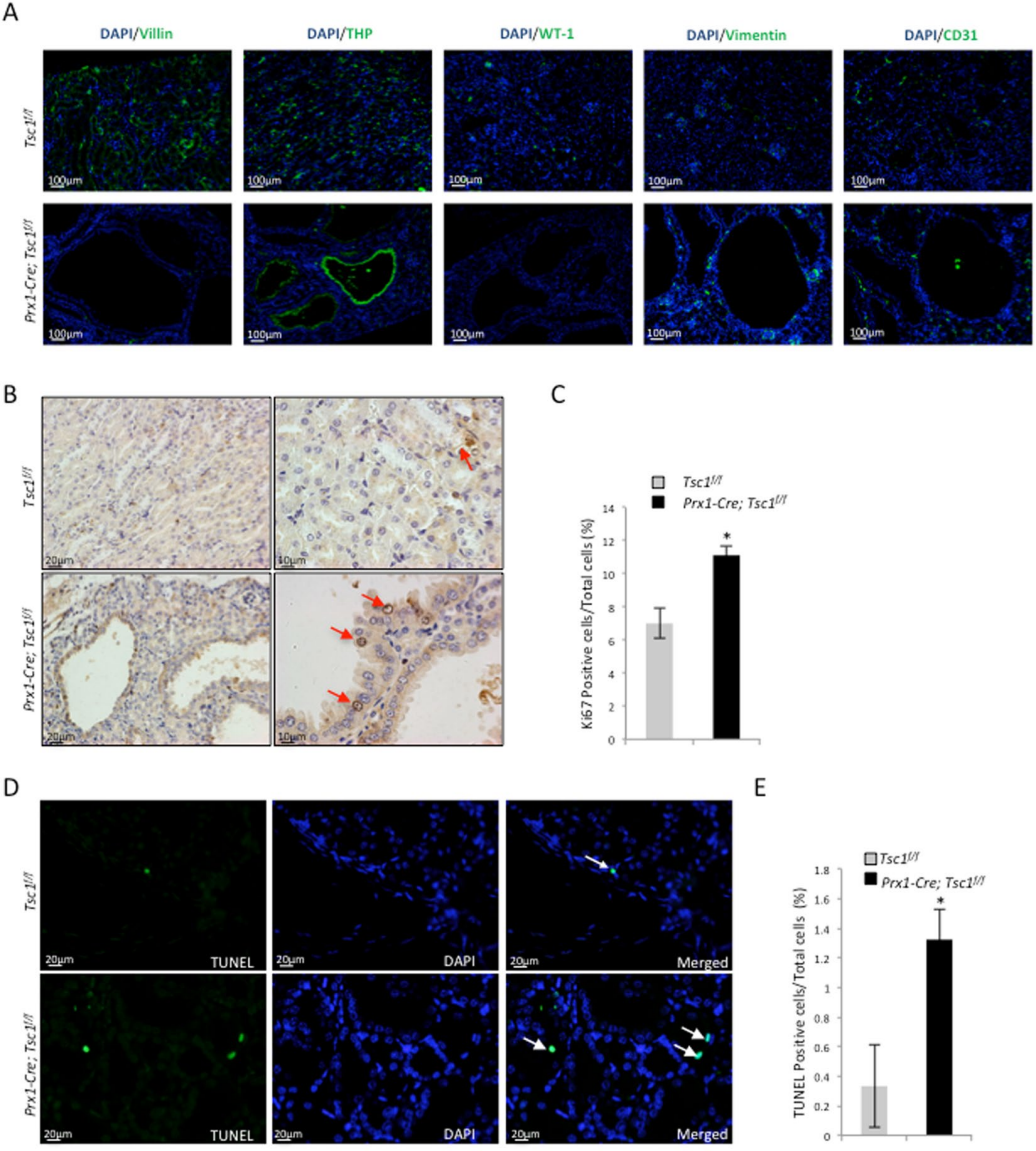
*Prx1-Cre; Tsc1*^*f/f*^ mouse kidney samples showed an increase in proliferating cells and apoptotic cells compared to control mice. (**A**) Immunofluorescent staining for different renal cell markers at the cyst region of *Prx1-Cre; Tsc1*^*f/f*^ mouse revealed that the cysts were mainly positive for THP. (**B**) Representative images of Ki67-stained kidney sections. (**C**) Quantitative analysis of Ki67 positive cells. N = 3. *P < 0.05; **P < 0.001 when compared to the control mice. (**D**) Representative images of TUNEL stained kidney sections. (**E**) Quantitative analysis of TUNEL positive cells. N = 3. *P < 0.05; **P < 0.001 when compared to the control mice.

Immuno-staining revealed that all the cysts were positive only for THP, a loop of Henle marker, but negative for Villin, CD31, WT-1, and vimentin (Fig. 3A), indicating that the cysts were originated from the loop of Henle cells. Since Prx1 lineage cells were negative for THP themselves, these results suggest that Prx1 lineage cells may induce cyst formation via cell-cell contact or secretion of pro-growth factors.

### Rapamycin prevented cyst formation in *Prx1-Cre; Tsc1*^*f/f*^ mice

Ablation of *Tsc1* in Prx1-expressing cells resulted in mTORC1 activation. To assess the role of mTORC1 in cyst development and expansion, rapamycin was injected intraperitoneally into control and mutant mice for 4 weeks. Rapamycin injection resulted in a modest decrease in the body weights of control and mutant mice compared to vehicle-injected littermates (data not shown). At the end of the injection, the kidneys were harvested and analyzed. In rapamycin-treated *Prx1-Cre; Tsc1*^*f/f*^ mice, the morphological abnormalities of the kidney were rescued (Fig. 2B). A continuous scanning of H-E stained coronal sections of rapamycin-treated *Prx1-Cre; Tsc1*^*f/f*^ mouse kidney at 400 times magnification revealed only some tiny cysts (Fig. 2C and lower panels of Fig. 2E). The average size of the cysts and the cystic area/cortex tissue area were reduced in rapamycin-treated *Prx1-Cre; Tsc1*^*f/f*^ mice (Fig. 2F,G). Collectively, these results indicated that rapamycin inhibited cysts growth and alleviated kidney abnormalities of *Prx1-Cre; Tsc1*^*f/f*^ mice. The results also suggested that cystogenesis was largely caused by mTORC1 activation in *Prx1-Cre; Tsc1*^*f/f*^ mice.

### *Prx1-Cre; Tsc1*^*f/f*^ mice showed an increase in both cell proliferation and apoptosis

Kidney cyst formation can be caused by increased cell proliferation, decreased apoptosis, or both. We stained the kidney sections with anti-Ki67 antibodies and quantified the ratio of Ki67-positive nuclei to the total number of DAPI-stained nuclei (Fig. 3B). The result revealed an increase in the number of proliferative cells in the kidney of *Prx1-Cre; Tsc1*^*f/f*^ mice compared to controls (Fig. 3C). Quantitative analysis of TUNEL-stained sections revealed a higher percentage of TUNEL-positive cells, after normalized to DAPI-stained nuclei, in *Prx1-Cre; Tsc1*^*f/f*^ kidney than controls (Fig. 3D,E). These results suggest that enhanced cell proliferation underlies cystogenesis in *Prx1-Cre; Tsc1*^*f/f*^ mice, while increased apoptosis may be a secondary effect of increased cell proliferation.

### *Osx-Cre; Tsc1*^*f/f*^ mice showed cystogenesis that was alleviated by rapamycin

Osterix is a zinc finger protein essential for osteoblast differentiation from MSCs. Although it is considered as an osteoblast-specific transcription factor, it was also found expressed in the kidney^29^. The expression of Osterix is high at p4 but low at E17.5 or p21. The signals were detectable mainly in the juxtamedullary nephrons and proximal tubules at low levels and the descending loop of Henle at high levels. Moreover, heterozygous deletion of *Osx* resulted in increased IL6 expression and enhanced inflammatory responses upon renal injuries^30^. These results suggest that Osx is expressed in the kidney and plays a role in kidney injury and repair.

**Figure 4:**
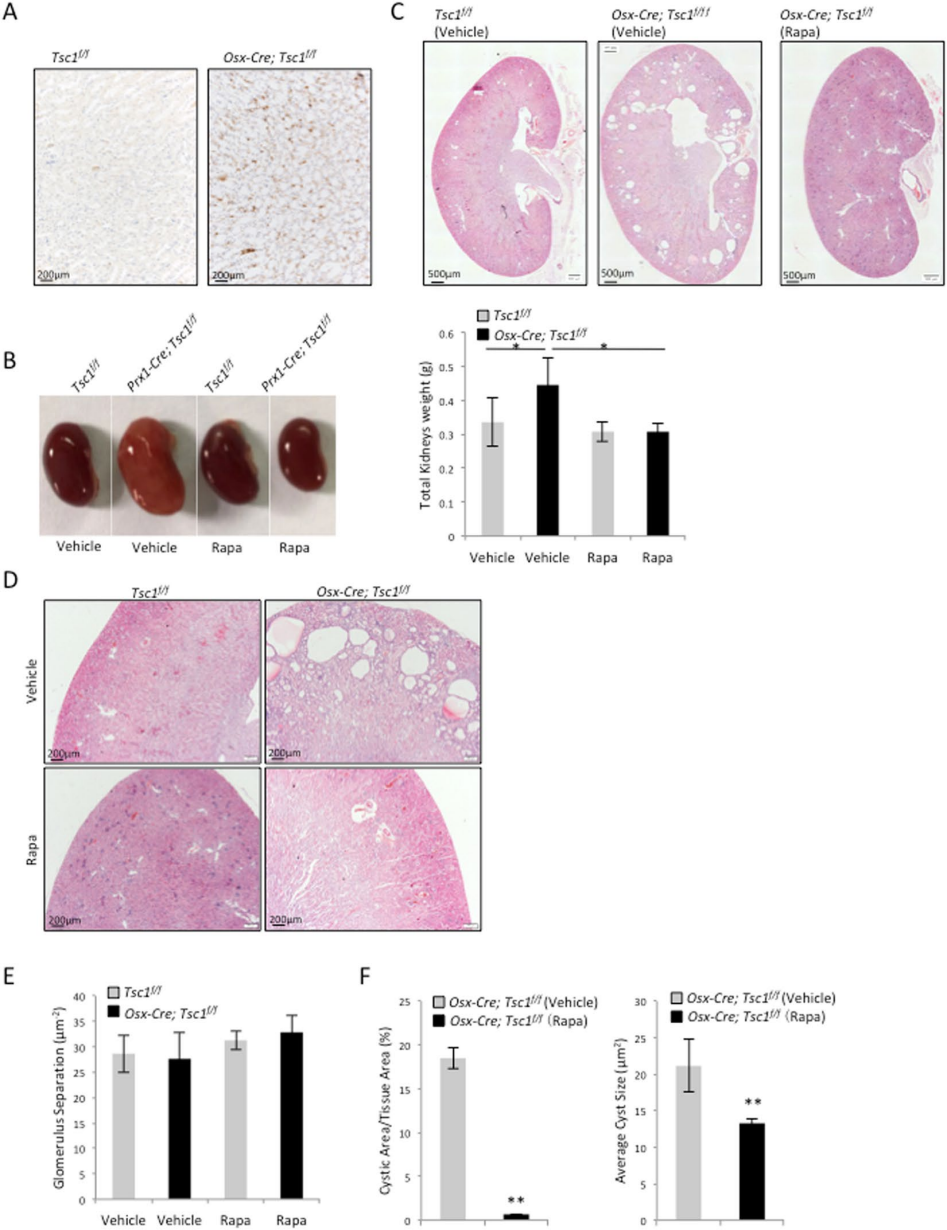
Conditional knockout of *Tsc1* in the Osx lineage resulted in the development of mTORC1-dependent cysts. (**A**) *Osx-Cre; Tsc1*^*f/ f*^ mouse kidney sections showed increased p-S6 signals. (**B**) *Osx-Cre; Tsc1*^*f/f*^ mice showed enlarged kidneys that were rescued by rapamycin. Left panel: the kidneys of different mouse lines. Right panel: the weights of the kidneys of different mouse lines. (**C**) H-E staining (4x) of the kidney sections of *Osx-Cre; Tsc1*^*f/f*^ and control mice treated with rapamycin or vehicle. (**D**) Images of kidney sections of *Osx-Cre; Tsc1*^*f/f*^ and control mice treated with rapamycin or vehicle. (**E**) Quantitative data of the cystic index of *Osx-Cre; Tsc1*^*f/f*^ and control mice treated with rapamycin or vehicle. The cystic index for control mice is 0. (**F**) Quantitative analysis of glomerulus separation. N = 3. *P < 0.05; **P < 0.001 when compared to the control mice.

**Figure 5:**
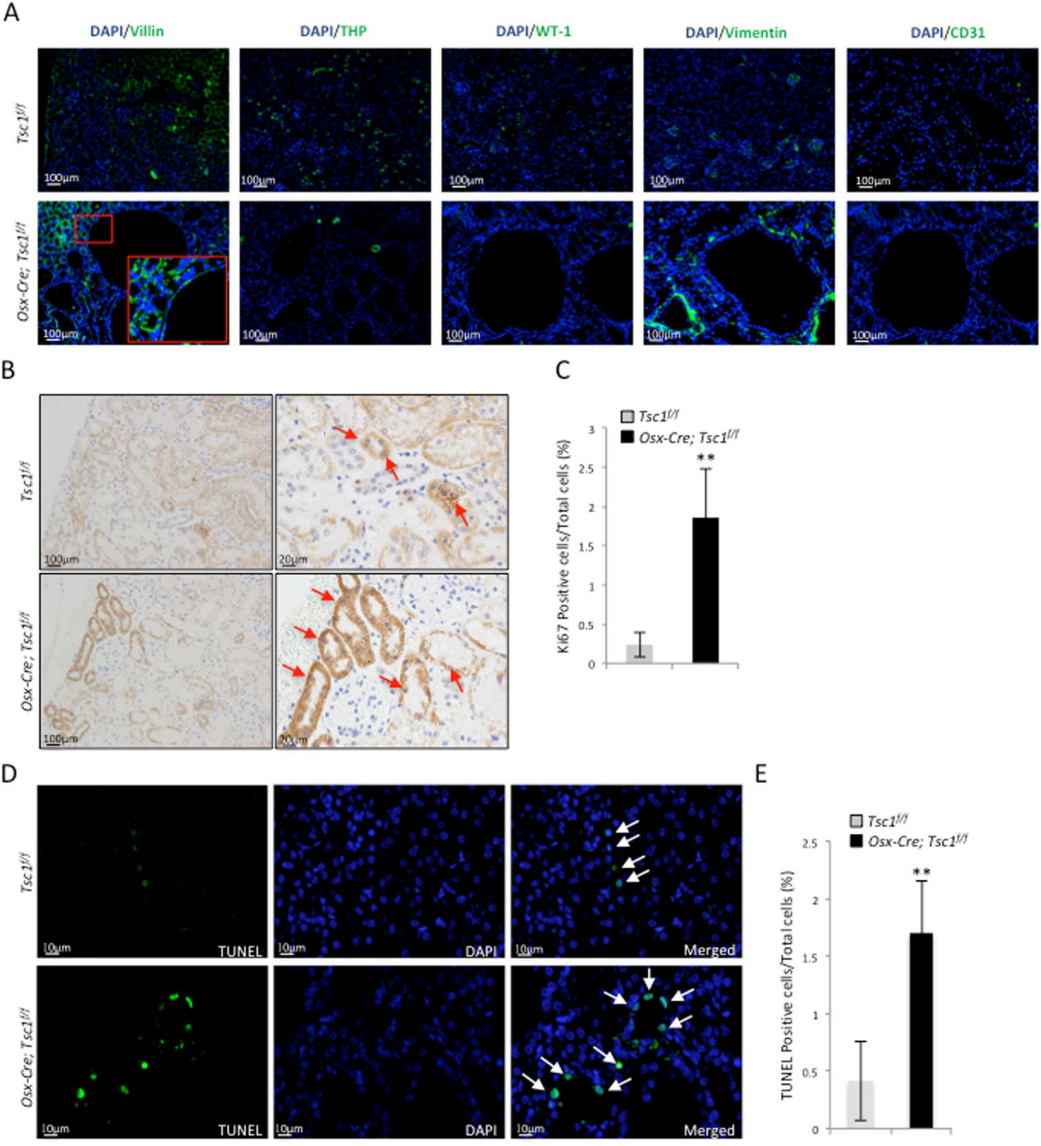
*Osx-Cre; Tsc1*^*f/f*^ mouse kidney samples showed increased proliferating cells and apoptotic cells compared to control mice. (**A**) Immunofluorescent staining for different renal cell markers at the cyst region of *Osx-Cre; Tsc1*^*f/f*^ mouse showed that the cysts were only positive for Villin. (**B**) Representative images of Ki67-staining kidney sections. (**C**) Quantitative analysis of Ki67 positive cells. (**D**) Representative images of TUNEL staining kidney sections. Arrows indicate apoptotic cells. (**E**) Quantitative analysis of TUNEL positive cells. N = 3. *P < 0.05; **P < 0.001 when compared to the control mice.

We generated *Osx-Cre; Tsc1*^*f/f*^ mice by crossing *Osx-Cre* mice with *Tsc1*^*f/f*^ mice. The up-regulation of p-S6 signals in *Osx-Cre; Tsc1*^*f/f*^ mice suggested an increase in mTOR activation in some of the renal cells (Fig. 4A). Adult *Osx-Cre; Tsc1*^*f/f*^ mice showed slightly larger kidney than the littermate controls (Fig. 4B, left panel). The average kidney weight of the *Osx-Cre; Tsc1*^*f/f*^ mice was greater than that of the control mice (Fig. 4B, right panel). H-E staining of the *Osx-Cre; Tsc1*^*f/f*^ mouse kidney sections revealed multiple cysts of varying sizes at the juxtamedullary cortex (Fig. 4C,D). The average cyst size and ratio of cyst area/tissue area were calculated and both indexes were greater than those of *Prx1-Cre; Tsc1*^*f/f*^ mice (Fig. 4E). However, no change in the glomeruli index was observed in *Osx-Cre; Tsc1*^*f/f*^ mice (Fig. 4F). Immunostaining revealed that the cysts were positive for Villin, a proximal tubular cell marker, but negative for THP, CD31, WT-1, or vimentin (Fig. 5A). Since Osx labels tubular cells, these results suggest that Tsc1 deletion in tubular cells leads to cyst formation. Note the morphology difference in cysts formed in *Prx1-Cre; Tsc1*^*f/f*^ mice and *Osx-Cre; Tsc1*^*f/f*^ mice.

Rapamycin was given to *Osx-Cre; Tsc1*^*f/f*^ mice and control mice daily via intraperitoneal injection for 4 weeks. Kidneys were harvested at the end of the injection. The kidneys of rapamycin-treated *Osx-Cre; Tsc1*^*f/f*^ mice appeared to be similar to the normal mice (Fig. 4B). H-E staining showed that rapamycin prevented the development of cysts in *Osx-Cre; Tsc1*^*f/f*^ mice (Fig. 4C,D). The average cyst size and the ratio of cystic area to total cortex tissue area were significantly reduced in rapamycin-treated *Osx-Cre; Tsc1*^*f/f*^ mice (Fig. 4E). These results suggested that the renal abnormalities observed in *Osx-Cre; Tsc1*^*f/f*^ mice were also caused by activated mTORC1.

### *Osx-Cre; Tsc1*^*f/f*^ mice showed an increase in cell proliferation and apoptosis

Cell proliferation and apoptosis in the kidney of the *Osx-Cre; Tsc1*^*f/f*^ mice were also analyzed using Ki67 immunostaining and TUNEL analysis, respectively. An increase in Ki67-positive cells and TUNEL-positive nuclei, after normalized to DAPI-stained nuclei, were observed in the kidney of the *Osx-Cre; Tsc1*^*f/f*^ mice compared to control mice (Fig. 5B–E). Again, these results suggested that elevated cell proliferation was involved in the development of cysts in *Osx-Cre; Tsc1*^*f/f*^ mice, similar to what was observed in *Prx1-Cre; Tsc1*^*f/f*^ mice (Fig. 3B–E).

### Dermo1-mediated ablation of *Tsc1* failed to produce renal phenotypes

**Figure 6:**
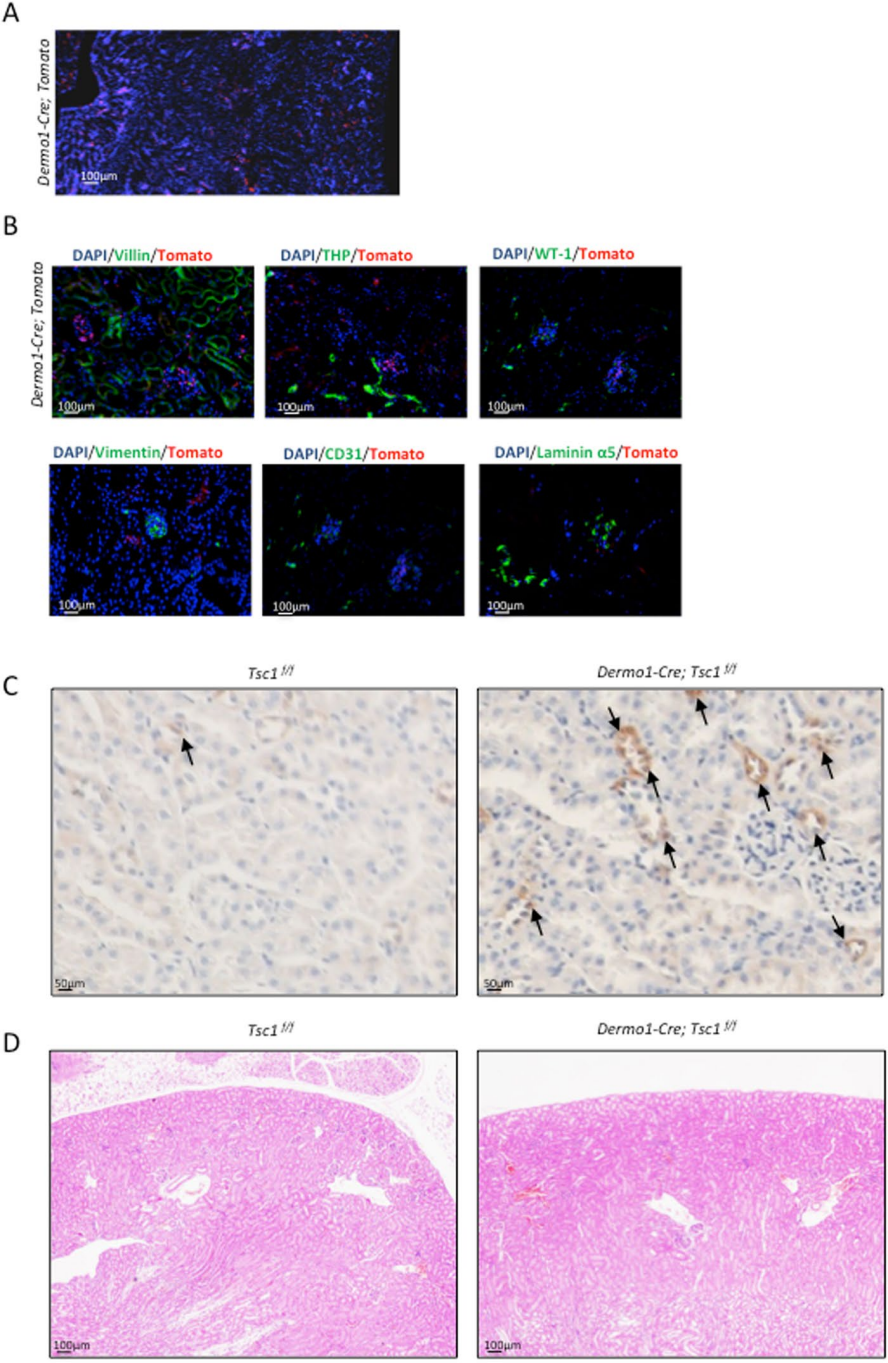
Conditional knockout of *Tsc1* in the *Dermo1* lineage did not result in cystogenesis. (**A**) Microscopic images of kidney sections of *Dermo1-Cre; tdTomato* mice showed that *Dermo1* could label tubular and glomerulus cells in mouse kidney. (**B**) Immunofluorescent staining on *Dermo1-Cre; tdTomato* mouse kidney sections revealed that Dermo1 linage renal cells were negative for the tested markers of different renal cell types. (**C**) *Dermo1-Cre; Tsc1*^*f/f*^ mouse kidney sections showed increased p-S6 signals. (**D**) H-E staining (4x) of the kidney sections of 2-month-old *Dermo1-Cre; Tsc1*^*f/f*^ and control mice.

Lastly, we tested the effects of mTOR signaling in Dermo1 lineage cells. A recent study showed that the deletion of *Tsc2* in Dermo1 lineage cells led to renal tubular epithelial hyperplasia and ploycystic kidney. The mice died at around 21 days after birth^31^. We first traced Dermo1 lineage cells in the kidney using *Dermo1-Cre; tdTomato* mice and found that the number of Dermo1-Cre lineage cells was in general less than that of Prx1 or Osx lineage cells. The signals were detected in both cortex and medulla of the kidney (Fig. 6A). Immuno-staining revealed that Dermo1 lineage cells were negative for Villin, THP, CD31, WT-1, Laminin α5, and vimentin (Fig. 6B), suggesting that Dermo1 lineage may constitute a group of renal stromal cells/fibroblasts.

We generated *Dermo1-Cre; Tsc1*^*f/f*^ mice. Immunostaining confirmed that the mutant mouse kidney had increased p-S6 signals (Fig. 6C). Surprisingly, the *Dermo1-Cre; Tsc1*^*f/f*^ mice were in good health. Histological analysis of the kidney failed to find cyst in 2- or 12-month-old mutant mice (Fig. 6D and data not shown). These results suggested significant functional differences between *Tsc1* and *Tsc2* in renal cell proliferation and apoptosis when their expression was under the control of *Dermo1* promoter.

## Discussion

The kidney nephrons contain several types of cells including podocytes, mesangial cells, and endothelial cells in the glomerulus, and tubular epithelial cells in the proximal, loop of Henle, and the distal tubules. In addition, there exist stromal cells/fibroblasts in the kidney, which are less well understood. The nephron cells including glomerulus cells are derived from the Six2-positive lineage whereas the interstitial stromal cells are derived from the Foxd1 lineage. In this study, we found that Prx1 and Dermo1 marked the cells in glomerulus and tubular regions that do not express markers for proximal tubular cells, loop of Henle cells, endothelial cells, mesangial cells, epithelial cells, or podocytes. While the identity of these cells awaits confirmation, these results suggest that they represent a group of stromal cells/fibroblasts. Moreover, Dermo1 lineage cells appear to be less abundant than the Prx1 lineage cells in the kidney. Our study thus identified additional genetic markers that could be used to label renal stromal cells. The relationships between these three markers and Six2 and Foxd1 warrant further investigation.

Although Prx1, Dermo1, and Osx label the MSC-osteoblast lineage in the bone marrow, they label renal cells types that are rather distinct. In bone marrow, MSCs labeled by Prx1 or Dermo1 are believed to be skeletal stem cells^32–35^, which are responsible for skeletal development and bone remodeling^36^, in a Osx-dependent manner^37,38^. Recent studies suggest that Dermo1 may mark only osteoprogenitors but not chondrocytes^39,40^. Thus Prx1, Dermo1, and Osx label the MSC-osteoblast lineage cells at different stages. However, our study shows that Dermo1 and Prx1 may label renal stromal cells, whereas Osx labels renal tubular cells, which are epithelial cells. The observation that *Tsc1* ablation mediated by Prx1-Cre, Dermo1-Cre, and Osx-Cre resulted in different renal phenotypes also suggest that these markers label different cells.

Recent studies have revealed that renal tubular cells possess the potential to trans-differentiate into osteoblast-like cells, which may contribute to the production of kidney stones under conditions such as calcium oxalate kidney stones and hyperoxaluric kidneys^41^. Our study reveal that MSC-osteoblast lineage markers could label the tubular cells. It is possible that some of renal tubular cells have the same origin as bone marrow MSCs and bear some features of osteoblasts. More importantly, Osx is known to promote calcification in bone tissues. The expression of Osx in renal tubular cells may participate in kidney stone formation.

TSC patients usually display overgrowth in brain, kidney, lung, eye and skin. We show here that *Tsc1* deficiency in Osx-labeled tubular cells induces cystogenesis in an mTORC1-dependent manner. The cysts in *Osx-Cre;Tsc1*^*f/f*^ mice are numerous and small and are located mainly in the renal cortex. Immunostaining reveals that these cysts are mainly derived from Villin-expressing proximal tubular cells. On the other hand, *Tsc1* deficiency in Prx1-labeled cells induces formation of unique cysts, which are rare but large. Immunostaining reveals that these cysts are mainly derived from THP-expressing loop of Henle cells. Since Prx1 lineage cells do not express THP, we conclude that Prx1 lineage cells induce cyst formation in a non-cell-autonomous manner. A previous study reported that ablation of *Pkd1*, encoding polycystin 1, in renal stromal cells caused progressive cystogenesis in the kidney^42^. These results, taken together, suggest that mutant *Tsc1* and *Pkd1* genes in renal stromal cells make an important contribution to cystogenesis. It is conceivable that *Tsc1* or *Pkd1* mutation in the stromal cells may lead to secretion of pro-growth factors to promote proliferation of renal tubular cells.

A surprising finding is that Dermo1-mediated *Tsc1* ablation failed to produce kidney cyst in young or old mice. This is in contrast to a recent study showing that Dermo1-mediated *Tsc2* ablation led to cyst formation^31^. Since the same *Dermo1-Cre* mouse line was used in both studies, the difference in the mouse phenotypes had to be due to the difference between *Tsc1* and *Tsc2*. Although *Tsc1* and *Tsc2* form a complex to inhibit the activation of mTOR signaling, they may have different activities that are independent of mTOR. Alternatively, their inhibitory effects on mTORC1 signaling may be different in magnitudes. Indeed, Kwiatkowski. *et al*. showed that renal tumors were lower in the *Tsc1* heterozygous mice compared to *Tsc2* heterozygous mice^2^. Moreover, *Tsc2* rather than *Tsc1* is the major driver for angiomyolipoma and renal cysts in TSC patients^43^, and *Tsc1* mutations are less common than *Tsc2* in TSC patients^44^. Interestingly, while we confirmed that *Tsc1* deficient tubular cells showed enhanced proliferation, we discovered an increase in the number of apoptotic cells in the absence of *Tsc1*, which may be caused by increased cell proliferation.

In summary, our study uncovers a population of cells in the kidney, stromal cells/fibroblasts, that are be labeled by a MSC marker, Prx1. Ablation of *Tsc1* in these cells causes cyst from loop of Henle, which is different from the cyst caused by ablation of *Tsc1* in Osx-labeled tubular cells in morphology and origin. These findings suggest stromal cells contribute to cyst formation in TSC patients.

## Materials and Methods

### Animals

The *Prx1-Cre*, *Osx-Cre*, and floxed *Tsc1* (*iDTR*, *tomato*) mouse lines were originally generated from the Jackson Laboratory. All mice used were crossed and housed in a specific-pathogen-free (SPF) mouse facility under standard conditions in Bio-X Institutes, Key Laboratory for the Genetics of Developmental and Neuropsychiatric Disorders, Shanghai Jiao Tong University, Shanghai, China.

All animal procedures were carried out following the recommendations from the National Research Council Guide for the Care and Use of Laboratory Animals, with the protocols being approved by the Institutional Animal Care and Use Committee of Shanghai, China [SYXK (SH) 2011-0112]. Unless otherwise specified, mice at the age of 2 months were sacrificed for individual experiment.

### Histology, Immunohistochemistry and Immunofluorescent microscopy

Kidneys were harvested immediately after the mice were sacrificed and rinsed in phosphate-buffered saline (PBS) twice before fixed in 4% freshly made paraformaldehyde (PFA). For histological and immunohistochemistry studies, kidneys were embedded in paraffin and sectioned at 5 μm thickness. H-E staining was carried out according to the standard protocol and images were scanned by Olympus DP80 (Olympus Microsystems, San Jose, CA, USA), and photographed by Olympus DP72 (Olympus Microsystems, USA).

For immunofluorescent microscopy, the fixed kidneys were dehydrated by incubating in increasing concentrations of sucrose solutions before being embedded in OCT. The snap-frozen tissues were kept at −80 °C until sectioned. Samples were sliced into 5 μm thickness sections and stained with ProLong Gold DAPI (Life Technologies, New York, USA). Images were taken and analyzed using the Olympus DP72 microscope.

For immunohistochemistry analysis, the tissue sections were deparaffinized in xylene, rehydrated and permeabilized with 0.1% Triton X-100 for 20 mins at room temperature. They were treated with citrate buffer in a pressurized oven for antigen retrieval. Subsequently, the slides were blocked with 10% goat serum in 1x PBS for 1 hour, incubated with specific primary antibody overnight at 4 °C, rinsed in 1x PBS, then incubated with 1% secondary antibody (diluted in 1x PBS) for 1 hour at 37 °C. After brief washing in 1x PBS, slides were incubated in 1% SABC solution for 1 hour at 37 °C.

### Renal Cyst Index Measurement

Both kidneys were excised, de-capsulated and the weight was combined. Coronal sections of the inner part of kidney were used for renal cyst index calculation. H-E stained sections were imaged at 100x magnification and used to count the number of glomerulus and cysts, the areas of cysts, renal cortex and the whole kidney. Glomerulus separation was defined as the ratio between the number of glomerulus and renal cortex. The cystic index was defined as the ratio between the combined area of cysts and the area of entire kidney.

### Cell apoptosis analysis

*In Situ* Cell Death Detection Kit (Roche, Basel, Switzerland) was used to examine cell apoptosis. De-waxed and rehydrated kidney sections were incubated with proteinase K working solution (20 µg/ml) for 20 mins at room temperature. The slides were rinsed in 1x PBS and the area surrounding the samples was dried. The TUNEL reaction mixture was added to the tissue slides and they were incubated in a humidified chamber for 1 hour at 37 °C. After the incubation, the slides were rinsed and the imaged directly under a Nikon ECLIPSE Ti fluorescence microscope (Nikon, Tokyo, Japan).

### Rapamycin Treatment

Rapamycin (3 μg/g body weight in 500 ng/µl) or vehicle at the same volume was injected daily into mouse abdomen for 4 weeks. Mice were sacrificed three days after the last injection.

### Statistical Analysis

All experiments were repeated at least three times and the averaged data ± standard deviation (SD) were presented. Students’ unpaired *t*-tests were performed to compare data between groups and the difference was considered significant if the p value equals or was less than 0.05.
